# Rural Trends in Diagnosis and Services for Autism Spectrum Disorder

**DOI:** 10.3389/fpsyg.2017.00590

**Published:** 2017-04-20

**Authors:** Ligia Antezana, Angela Scarpa, Andrew Valdespino, Jordan Albright, John A. Richey

**Affiliations:** ^1^Department of Psychology, Virginia Tech, BlacksburgVA, USA; ^2^Virginia Tech Center for Autism Research, BlacksburgVA, USA

**Keywords:** autism spectrum disorder, rural, diagnosis, screening, intervention, telehealth

## Abstract

Rural communities face significant challenges regarding the adequate availability of diagnostic-, treatment-, and support-services for individuals with autism spectrum disorder (ASD). Specifically, a variety of factors, including geographic distance between families and service providers, low reliance on health care professionals, and cultural characteristics, contribute to the diminished availability and utilization of services. Together, these factors lead to risks for delayed ASD screening and diagnosis, yielding lower educational and functional outcomes. The purpose of this review is to outline the specific diagnosis and treatment barriers that affect individuals with ASD and their families in rural settings. Telehealth feasibility and efficacy research is also reviewed, suggesting that telecommunication services may offer an inroad for addressing the specific service barriers faced by rural communities. Together, the current review identifies specific needs for both research and support services that address the specific access barriers characteristic of rural settings.

## Introduction

It is widely accepted that early diagnosis and intervention are important factors for improving functional outcomes in children diagnosed with autism spectrum disorder (ASD). Although similar rates of ASD prevalence are reported in both rural (0.9%) and urban (1.0%) areas (Mohamed et al., 2016), individuals in rural communities, report limited access to the types of resources required for timely and appropriate identification and intervention services (Cohen and Hesselbart, 1993; Slade, 2003; Green et al., 2013; Gona et al., 2016). There are specific factors which contribute to barriers in diagnosis and services for affected individuals and families in rural settings. Rural settings are characterized by lessened availability of services per capita and low socioeconomic status and education levels (Hartley, 2004). Moreover, in addition to a diminished presence of and access to minimally adequate care for rural areas, there is a lack of evidence-based practices for identifying and providing services for individuals with ASD (Rhoades et al., 2007). This combination of factors contributes to a variety of unfavorable outcomes for affected children who live in rural locations, such as delays in developmental screening and diagnosis as well as fewer available interventions, which can lead to comparatively worse educational and functional outcomes (Mandell et al., 2005; Scarpa et al., 2013). The purpose of this review is to synthesize the available literature examining three main themes in rural ASD populations (1) pathways to identification and services (2) differential individual and cultural characteristics of affected rural children and (3) barriers of service implementation in rural communities.

## Pathways to ASD Identification and Services

Rural settings present specific disparities and concrete challenges to families of children with ASD (King et al., 2010; Ws et al., 2015). Chief among these is limited physical access to resources due to geographic distance. Specifically, parent-report data from the 2011 Survey of Pathways to Service and Diagnosis (SPSD) has demonstrated that children with a current diagnostic status of ASD who live in rural versus urban areas are more likely to report experiences of significant difficulties and delays due to lack of available services (rural: 35%; urban: 23%). For rural communities, these geographic circumstances and sparse availability of services prompt the primary care system to rely on other entities such as schools for ASD identification and services.

Schools in rural settings have been suggested to function as a “*de facto* mental health system” for youth (Burns et al., 1995). Consistent with this suggestion, findings from the 2011 SPSD indicate that while parents of children with ASD report similar rates of speaking with school personnel about their concerns, parents in rural areas report lower rates (88%) of speaking to health care providers about their concerns compared to urban parents (93%). Moreover, there are significant differences in rural versus urban parent-report on how doctors respond to concerns. For example, doctors are more likely to suggest that the parent discuss the concern with the school for rural (40%) versus urban (28%) areas (Centers for Disease Control and Prevention et al., 2011). Altogether, these data reflect increased dependence on the school system for access to services and screening in rurally situated families, and comparatively less reliance on health care providers as compared to urban families.

Despite the observation that parents of children with ASD who live in rural areas report a high reliance on schools for referrals and support, it is also known that children who are first identified through the school system are the least likely to receive services (Farmer et al., 2003). As a result, although the school system plays a pivotal role in initiating pathways to services and diagnosis for rural communities, it is likely that children who are first flagged for ASD in schools are less likely to be properly linked to support entities for diagnosis and services. The increased geographic distance between practitioners and affected individuals for rural areas further compounds this specific demand for strong and direct links between practitioners and central community areas (e.g., school systems, churches) in order to identify individuals with ASD in a timely manner and further connect families to proper services (e.g., special services, mental health services).

## Differential Individual and Cultural Characteristics of Affected Rural Children

It is estimated that 1 in 68 children, by the age of 8 years, is identified as having an ASD diagnosis in the United States (Christensen et al., 2016). The prevalence of ASD in rural versus urban communities reveal a number of important patterns. Data from (National Survey of Children’s Health [NSCH], 2007), a population based epidemiological sample, demonstrate that there are similar rates of ASD in both rural and urban areas. Thus, the observation of differential rates of diagnosis that are reported in non-population-based samples may actually be partially an artifact of better, and more densely concentrated resources in urban centers. In support of this, one study examining rates of ASD across a number of countries (Japan, the United States, France, and Sweden) found that urbanicity explained 53% of the variance in prevalence. Specifically, urban areas reported rates that were over 2.5 times greater than rural areas (Williams et al., 2006). Similar trends have been found in China and Denmark (Wan et al., 2013; Vassos et al., 2016). Together, these data support the hypothesis that, while prevalence rates may not differ, more densely populated areas have greater ASD awareness (Palmer et al., 2005). This lack of ASD awareness is prevalent not only across communities, but with school and health providers as well.

Further, reduced awareness and diminished screening in rural areas may lead to a certain segment of ASD cases to be undetected, specifically cases in which there is no co-occurring intellectual or language impairment. As such, these ‘higher-functioning’ ASD cases may be more likely to be missed or misdiagnosed. This notion is supported by findings of high rates of co-occurring ASD and intellectual disability in rural compared to urban areas of the UK, as well as by the increase of residential facilities for ASD and intellectual disability in rural areas (Kiani et al., 2013). On a more promising note, these findings also suggest that there are some systems in place for detecting and accommodating individuals with ASD with co-occurring intellectual or language disability in rural areas, although more work is needed in identifying and providing services for those without such impairments. Taken together, these findings suggest that improved resources for identifying ASD in rural communities are critical and may also need to better target ASD without intellectual or language impairments.

In addition to the above-noted individual characteristics and relative lack of ASD awareness (i.e., knowledge of the disorder and services) that impact detection in rural areas (Wallace et al., 2012; Hoogsteen and Woodgate, 2013a), there are also cultural characteristics that distinguish these areas and may contribute to the diminished identification and use of services for affected individuals with ASD (Strasser, 2003). Specifically, there are significant disparities in socioeconomic status and levels of education in rural areas compared to urban, with marked lower socioeconomic status and level of education in rural areas (Blumenthal and Kagen, 2002; Hartley, 2004). The geographic distance from central resources in addition to low socioeconomic status, may create a specific burden for affected families in rural areas, as travel costs can deter them from seeking services (Ashburner et al., 2016). Moreover, low parental education level may additionally contribute to reduced ASD knowledge and identification, as parents with higher education tend to get diagnosed earlier (Fountain et al., 2011). Rural culture is described as close-knit, with traditionalistic views, and a high regard for self-sufficiency, self-reliance and independence (Strasser, 2003). This cultural independence and apprehension of outside professionals can result in avoidance of initial screening and formal services. Further, it may be difficult for practitioners to gain trust and effectively communicate with individuals in these areas. Finally, lack of awareness by both professionals and communities adds an additional complication to the dual issues of identification and services (Rhoades et al., 2007; Holt and Christensen, 2013; Hoogsteen and Woodgate, 2013b). The combination of these factors may collectively worsen outcomes for affected individuals and families.

## Implementation of ASD Services in Rural Settings

In general, poor availability and implementation of mental health services for children has been reported for rural areas (Cohen and Hesselbart, 1993; Slade, 2003; Green et al., 2013; Cummings et al., 2015), however, there is little research examining the specific barriers that prevent children with ASD and their families from benefitting fully from services available in rural settings. In this section, we outline recently identified barriers to implementation, and we suggest specific solutions that may increase the availability of effective screening and intervention strategies.

Early screening for ASD is a catalyst for early diagnosis, intervention, and greater outcomes (Committee on Children With Disabilities, 2001; Volkmar et al., 2014). Screening for ASD might ideally start in the pediatrician’s office, but there may also be a lack of awareness from health professionals and lack of specialized professionals in the area (Elder et al., 2016). Further, very few service providers in rural areas specifically screen for ASD (Janvier et al., 2016), putting these communities at a greater risk of not being identified. These problems further contribute to the delay in diagnosing a child for these areas. Screeners for ASD may have poor reliability in some rural groups, especially for individuals with minority status or low education (Scarpa et al., 2013), and it has been suggested that some screening questions may not be appropriate (e.g., “Does your child ever pretend, for example to talk on the phone or take care of a doll or pretend other things?”) for the population due to social demographic variables (Mohamed et al., 2016). Cultural variation in rural settings further magnifies potential screening biases suggesting that screening measures may need to be adapted for rural populations to establish reliability. Delayed identification of affected individuals may lead to worse prognostic outcomes and further delays in school services. Future research to identify culturally sensitive and appropriate measures for rural populations is needed.

School service needs for youth with ASD in the United States are outlined by requirements within the Individuals with Disabilities Education Act (IDEA, 2004) and the National Research Council (NRC, 2001). In the United States, a multidisciplinary, team-developed plan also known as an individualized education plan (IEP) is required for every child receiving special education services (IDEA, 2004). Rural youth with ASD are at a higher risk of being missed and not receiving federally mandated levels of education services, due to the fact that services are less proliferate and more difficult to access. It is known that the quality of a child’s IEP (i.e., measurable social, communication and learning goals that can be monitored over time) is one of the greatest predictors of educational outcome (Ruble and McGrew, 2013). Rural settings are at risk of worse educational outcomes if practitioners and school personnel lack ASD awareness (e.g., may not suggest appropriate recommendations and services), and health care providers cannot aid in advocating for individuals (e.g., incapable of attending IEP meetings due to barriers related to time and cost). Further, although speech and language therapy are school-based services that are frequently reported for children with ASD, school-based social skills interventions are less likely to be formally implemented (White et al., 2007). For rural youth with ASD, this absence of school-based social skill intervention may mean poorer social outcomes, as affected individuals are less likely to pursue outside services due to geographic barriers. Altogether, the implementation of school-based services for affected rural youth is rooted in developing a high quality IEP with clear goals, including social skills goals, as these are central to ASD outcomes.

As a response to the problems presented by geographic distance, a productive line of research has focused on telehealth (e.g., telemedicine, e-health) and similar interactive strategies, in order to administer assessment and in some cases intervention to physically remote communities. Telehealth is the delivery of health services through the use of information and communication technologies; this method has been increasingly studied as a means to deliver services for individuals with ASD (Ashburner et al., 2016). Telehealth services provide rural communities with a cost- and time-effective way for families to pursue diagnosis and interventions. In the United States, some evidence-based telehealth services for ASD can be delivered nationally, such as applied behavior analysis (ABA) and cognitive behavioral therapy (CBT). It should be noted that these interventions vary in cost and insurance coverage. Further, several telehealth-based applications are being developed with video and survey capabilities for screening of ASD (e.g., Duke University’s Autism & Beyond application) indicating a future aid for screening in rural areas. Preliminary data has shown that telehealth-based diagnostic services using autism diagnostic measures may be a viable method for assessing ASD. One study found no difference in reliability of diagnostic accuracy between ‘in person’ and ‘video conference’ conditions (Reese et al., 2013), although it should be noted that this study did not focus on fidelity but rather solely whether both clinicians agreed on ASD diagnosis. Telehealth-based interventions for ASD have also been studied with ABA with acceptable fidelity and successful treatment of behavior problems (Suess et al., 2013; Lindgren et al., 2016). A targeted social communication treatment using ABA principles has also been delivered via telehealth in rural areas as a pilot study. Although data regarding fidelity and outcomes is not yet available, high rates of parent engagement and satisfaction have been reported (Ingersoll and Berger, 2015). Further, telehealth IEP consultations for ASD have been noted to improve IEP quality and educational outcomes (Ruble et al., 2013). Additionally, a recent pilot study used telehealth to deliver a modified manualized CBT for co-occurring anxiety with ASD (“Facing Your Fears”) that appears to be effective in reducing symptoms of anxiety (Reaven et al., 2012). Investigators also found acceptable feasibility rates as noted by the high session attendance (94%), and mean satisfaction ratings for both parents (93%) and children (89%), and acceptable fidelity from clinicians (92%). Support for preliminary efficacy was further underscored by the significant improvement in parent report of youth anxiety for the telehealth group compared to waitlist controls (Hepburn et al., 2016). Although there are several evidence-based services and practices that have been supported for ASD via telehealth, with major benefits (i.e., lower cost, improved access), there continue to be barriers to the implementation of telehealth services. Of note, although several ASD-specific means of telehealth services have been supported for through grant-funded research, access to such services may diminish once grants end. Further, insurance coverage and service reimbursement is limited depending on one’s state and location (Weinstein et al., 2014). Moreover, additional barriers still remain; for example, many remote rural communities may not have easy access to the internet or to computers for this type of intervention.

Together the studies listed above highlight several barriers in the implementation of ASD diagnosis and services. Although telehealth services may be a potential cost- and time-effective avenue for receiving diagnosis and treatment for families with ASD, the practicality of this implementation warrants further research. Moreover, professional expertise may be lacking because of the reduced populations and job capacity in these communities. Finally, characteristics of the service settings themselves may cause complications, such as administrative support and learning climate for evidence-based practices (Drahota et al., 2012). It is likely that increases in numbers as well as education for practitioners, school professionals, and community members in rural areas may aid in the early identification and access to appropriate services for these families.

## Conclusion

The barriers noted throughout the ASD screening, diagnosis, and intervention literature for rural areas reflect the need for new adaptations of current measures, greater ASD awareness, and greater availability of affordable and accessible specialized services. Moreover, the systems in place for individuals with ASD in rural areas may lack specialization in identifying and treating higher functioning individuals with ASD, as more severe cases of ASD are less likely to be missed due to the level of impairment and distress the behaviors can cause the individual, family, and community. The combination of factors that characterize rural areas (i.e., geographic barriers, lack of ASD awareness, cultural perspectives, low socioeconomic status, low educational attainment), contribute to the risk of individuals with ASD being missed or misdiagnosed in these communities. Although services for youth with ASD in rural areas are lacking, telehealth services show a promising shift for reliable, affordable, and convenient health care for a hard to reach population. Yet, multiple other barriers remain, making it critical for future research to understand the specific needs of underserved populations and ways to successfully address them.

## Author Contributions

LA, AS, AV, JA, and JR were involved in the conception, drafting, and revisions of the article.

## Conflict of Interest Statement

The authors declare that the research was conducted in the absence of any commercial or financial relationships that could be construed as a potential conflict of interest.
